# Association of *IREB2 *and *CHRNA3 *polymorphisms with airflow obstruction in severe alpha-1 antitrypsin deficiency

**DOI:** 10.1186/1465-9921-13-16

**Published:** 2012-02-22

**Authors:** Woo Jin Kim, Alice M Wood, Alan F Barker, Mark L Brantly, Edward J Campbell, Edward Eden, Gerard McElvaney, Stephen I Rennard, Robert A Sandhaus, James M Stocks, James K Stoller, Charlie Strange, Gerard Turino, Edwin K Silverman, Robert A Stockley, Dawn L DeMeo

**Affiliations:** 1 Department of Internal Medicine, Kangwon National University, Chuncheon, South Korea; 2 School of Clinical and Experimental Medicine, University of Birmingham, Birmingham, UK; 3 Oregon Health and Science University, Portland, OR, USA; 4 University of Florida, Gainesville, FL, USA; 5 Intermountain Health Care, Provo, and Heredilab, Inc, Salt Lake City, UT, USA; 6 St. Luke's/Roosevelt Hospital, New York, NY, USA; 7 Beaumont Hospital, Dublin, Ireland; 8 University of Nebraska, Omaha, NE, USA; 9 National Jewish Health, Denver, CO, USA; 10 University of Texas at Tyler, Tyler, TX, USA; 11 Cleveland Clinic, Cleveland, OH, USA; 12 Medical University of South Carolina, Charleston, SC, USA; 13 St. Luke's/Roosevelt Hospital, New York, NY, USA; 14 Channing Laboratory and the Division of Pulmonary and Critical Care Medicine, Brigham and Women's Hospital, and Harvard Medical School, Boston, MA, USA; 15 Lung Investigation Unit, University Hospitals Birmingham, Birmingham, UK

**Keywords:** *CHRNA3*, Chronic obstructive pulmonary disease, Genetic association analysis, Genetic modifiers, *IREB2*

## Abstract

**Background:**

The development of COPD in subjects with alpha-1 antitrypsin (AAT) deficiency is likely to be influenced by modifier genes. Genome-wide association studies and integrative genomics approaches in COPD have demonstrated significant associations with SNPs in the chromosome 15q region that includes *CHRNA3 *(cholinergic nicotine receptor alpha3) and *IREB2 *(iron regulatory binding protein 2).

We investigated whether SNPs in the chromosome 15q region would be modifiers for lung function and COPD in AAT deficiency.

**Methods:**

The current analysis included 378 PIZZ subjects in the AAT Genetic Modifiers Study and a replication cohort of 458 subjects from the UK AAT Deficiency National Registry. Nine SNPs in *LOC123688, CHRNA3 *and *IREB2 *were selected for genotyping. FEV_1 _percent of predicted and FEV_1_/FVC ratio were analyzed as quantitative phenotypes. Family-based association analysis was performed in the AAT Genetic Modifiers Study. In the replication set, general linear models were used for quantitative phenotypes and logistic regression models were used for the presence/absence of emphysema or COPD.

**Results:**

Three SNPs (rs2568494 in *IREB2*, rs8034191 in *LOC123688*, and rs1051730 in *CHRNA3*) were associated with pre-bronchodilator FEV_1 _percent of predicted in the AAT Genetic Modifiers Study. Two SNPs (rs2568494 and rs1051730) were associated with the post-bronchodilator FEV_1 _percent of predicted and pre-bronchodilator FEV_1_/FVC ratio; SNP-by-gender interactions were observed. In the UK National Registry dataset, rs2568494 was significantly associated with emphysema in the male subgroup; significant SNP-by-smoking interactions were observed.

**Conclusions:**

*IREB2 *and *CHRNA3 *are potential genetic modifiers of COPD phenotypes in individuals with severe AAT deficiency and may be sex-specific in their impact.

## Background

Chronic obstructive pulmonary disease (COPD) is a complex disease characterized by persistent airflow limitation. COPD risk likely results from the cumulative effect of environmental factors (especially cigarette smoking), genetic factors, and gene-by-environment interactions [1]. Alpha-1 antitrypsin (AAT) deficiency, typically caused by homozygosity for the Z allele at the AAT gene (*SERPINA1)*, is a proven genetic cause of COPD. However, the development of COPD and emphysema in subjects with AAT deficiency is highly variable and is likely influenced by modifier genes and environmental factors [2-4].

Spirometric measurements of pulmonary function are widely used phenotypes in evaluating AAT deficient subjects and monitoring lung function decline [5]; CT scan assessments for emphysema have been used as additional intermediate phenotypes of COPD to overcome some of the heterogeneity inherent in spirometric classifications alone. Familial aggregation studies of pulmonary function have suggested additional modifier genes in AAT deficiency subjects [6,7]. A few potential AAT candidate modifier genes, including *NOS3 *[8], *GSTP1 *[9], *TNF *[10], and *IL10 *[11], have been reported so far, but these results have not been consistently replicated.

Genome-wide association (GWA) studies have revolutionized the identification of susceptibility genes for complex diseases. Three recent GWA studies showed that SNPs in a region of chromosome 15q25 were significantly associated with lung cancer; several nicotinic acetylcholine receptor genes, including *CHRNA3 *and *LOC123688*, are located in this region [12-14]. A genome-wide association (GWA) study in COPD also showed significant associations between COPD susceptibility and SNPs in this region [15]. This region was also associated with airflow obstruction and emphysema [16,17]. Interestingly, in addition to nicotinic acetylcholine receptor genes, this region also includes *IREB2 *(iron regulatory binding protein 2). *IREB2 *was identified as a potential COPD susceptibility gene using an integrative genomics approach with gene expression analysis of lung tissue samples followed by genetic association analysis [18]. We hypothesized that SNPs in this chromosome 15q region may be modifiers of intermediate phenotypes of COPD in subjects with severe AAT deficiency.

## Methods

### Study subjects

The current analysis included 378 subjects with severe AAT deficiency (protease inhibitor [PI] ZZ) from 167 families in the AAT Genetic Modifiers Study. Ascertainment of eligible sibling pairs was based on homozygosity for the Z allele at the *SERPINA1 *locus as previously described [19]. Pre- and post-bronchodilator study spirometry testing was performed according to American Thoracic Society (ATS) standards as described previously [19]. Percent predicted values for FEV_1 _were calculated using equations of Crapo and colleagues for Caucasian subjects [20]. The FEV_1_/FVC ratio was analyzed using unadjusted values. Pack-years of cigarette smoking were calculated by multiplying the number of years smoked by the average number of daily cigarettes smoked, divided by 20. All participants provided written informed consent, and the study protocol was approved by individual Institutional Review Boards at each of the participating clinical centers (Partners IRB, 2001P001237). 458 unrelated Caucasian subjects from the UK AATD National Registry were also genotyped. Approval for the study was given by the local ethics committee. All patients had a serum alpha-1 antitrypsin (AAT) level of < 11 μM and PI ZZ genotype confirmed by allele-specific PCR (Heredilab, Salt Lake City, Utah, USA). None of the UK subjects had ever received AAT augmentation therapy. A full clinical assessment including smoke exposure, presence of chronic bronchitis (defined as a productive cough for at least 3 months in at least 2 consecutive years [21]), lung function testing and high resolution CT scanning of the chest was undertaken, as described previously [22]. The presence of emphysema was determined by the appearance of the scan and density mask analysis of slices at the level of the aortic arch (representing the upper zone) and the inferior pulmonary vein (representing the lower zone) using a threshold of -910 Hounsfield Units (HU). This HU threshold has been validated against physiological measures in AATD [22]. Patients whose voxel index exceeded values seen in normal subjects in either zone [23] were classified as having emphysema.

### Genotyping

Two SNPs (rs8034191 and rs1051730) in chromosome 15 were selected from the previous GWA in COPD [15]. Additionally, 7 SNPs in *IREB2 *were selected using pairwise linkage disequilibrium (LD)-tagging in Tagger with a minimum minor allele frequency of 0.05 and r^2^-threshold of 0.8 [24]. SNPs were genotyped using Sequenom (San Diego, CA) assays in the AAT Genetic Modifiers Study. All family data were evaluated for familial inconsistencies using the PEDCHECK program [25].

In UK AATD National Registry study, genotyping was carried out using TaqMan^® ^technologies (Applied Biosystems, UK) on an ABI7900 HT for 3 SNPs associated in the test dataset (rs2568494, *IREB2*; rs8034191, *LOC123688*; rs1051730, *CHRNA3*). All genotyping assays were pre-validated by the suppliers, and all plates included appropriate negative controls.

### Statistical analysis

Hardy-Weinberg equilibrium was assessed using goodness of fit tests. Pre- and post-bronchodilator FEV_1 _percent of predicted and pre- and post-bronchodilator FEV_1_/FVC ratio were analyzed as quantitative phenotypes. Family-based association analysis was performed using PBAT software version 3.6 [26] assuming additive genetic models, adjusting for pack-years and pack-years^2 ^of cigarette smoking, under the null hypothesis of no linkage and no association in the AAT Genetic Modifiers Study. In addition to the overall model, we evaluated gender-stratified models and models that included a SNP-by-smoking or a SNP-by-gender interaction term. Haplotype analysis was performed using 8, 4, 3, 2 SNP adjacent sliding windows in PBAT.

In the UK AATD National Registry study, data were analyzed using SPSS (version 16, Chicago: SPSS Inc). Quantitative genetic association analysis was carried out for FEV_1 _and FEV_1_/FVC using general linear models, adjusting for age, gender and smoke exposure (as pack-years and pack-years^2^). Logistic regression models were used for the presence of emphysema or COPD (defined as post bronchodilator FEV_1_/FVC < 0.7) accounting for covariates as before. Additive models were assumed for all SNPs. Gender stratification and SNP-by-gender and SNP-by-smoking interaction analyses were also carried out, as in the test dataset.

## Results

### Demographic characteristics

The mean age of subjects was 52.2 years, and mean post-bronchodilator FEV_1 _was 65.9% predicted in the AAT Genetic Modifiers Study and 50.3 years and 53.8% predicted in the UK AATD National Registry, respectively (Table 1). Male subjects were 46% in AAT Genetic Modifiers Study and 59% in UK AATD National Registry, respectively. Three hundred and sixty-six subjects (79.8%) had emphysema in UK AATD National Registry.

**Table 1:** Baseline characteristics for PI ZZ individuals in the AAT Genetic Modifiers Study and the UK AATD National Registry

Characteristics	AAT Genetic Modifiers Study (*n *= 378)	UK national registry for AATD (*n *= 458)	*p *value
Age, years	52.2 ± 9.7	50.3 ± 10.4	0.01
Male sex (%)	173 (46%)	252 (55%)	< 0.0001
FEV_1_% predicted	65.9 ± 33.5	53.8 ± 32.2	0.0001
FEV_1_/FVC	0.551 ± 0.207	0.445 ± 0.194	< 0.0001
Pack-years for ever smokers	18.2 ± 14.5	15.9 ± 14.7	< 0.0001

Data are presented as means (± S.D.) in the AAT Genetic Modifiers Study and the UK national registry for AATD, unless otherwise noted

### Association analysis

There were no deviations from Hardy Weinberg equilibrium for any of the genotyped SNPs.

**Figure 1:**
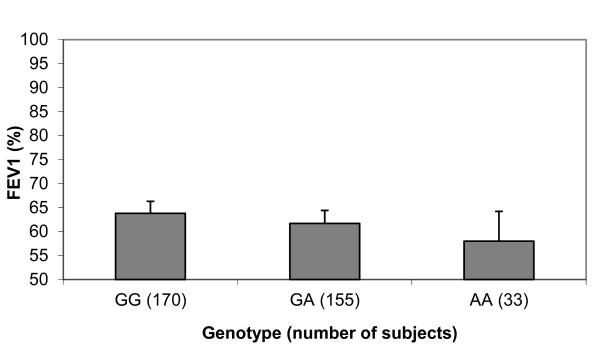
**FEV_1 _by *CHRNA3 *genotype (rs1051730) in the AAT Genetic Modifiers Study cohort**. Mean values (+ SEM) for a percent of predicted FEV_1 _are shown (*p *value = 0.02).

**Figure 2:**
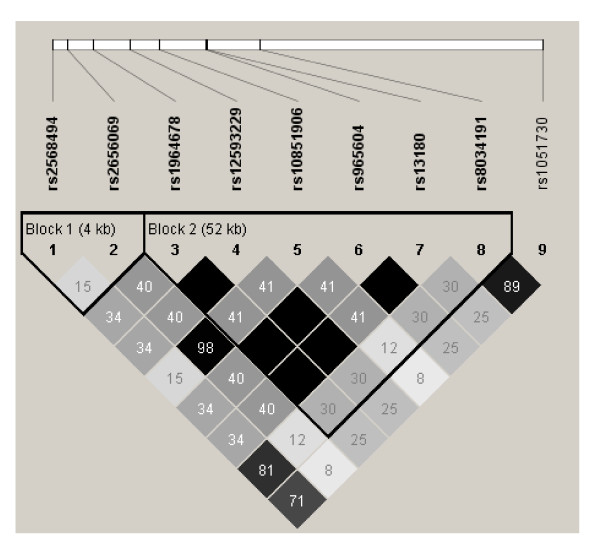
**Linkage disequilibrium (LD) among SNPs analyzed in chromosome 15. LD values are presented as r^2^**.

In the AAT Genetic Modifiers Study, three SNPs (rs2568494 in *IREB2*, rs8034191 in *LOC123688*, and rs1051730 in *CHRNA3*) were associated with pre-bronchodilator FEV_1 _percent of predicted (*p *< 0.05 Table 2 Figure 1). Two SNPs (rs2568494 and rs1051730) were associated with post-bronchodilator FEV_1 _percent of predicted and pre-bronchodilator FEV_1_/FVC ratio. One SNP (rs1051730) was associated with post-bronchodilator FEV_1_/FVC ratio (Table 2). Linkage disequilibrium (assessed with r^2^) between rs8034191 and the 1051730 was 0.9 (Figure 2). There was significant association only with a 2 SNP haplotype including rs8034191 and rs1051730 with pre- bronchodilator FEV_1_/FVC ratio (global test statistic; *p *= 0.05) using PBAT.

**Table 2:** Genetic association results between SNPs in chromosome 15 and lung function in the AAT Genetic Modifiers Study

*P*-Values for Different Phenotypes							
**Gene**	**SNP**		**MAF**	**pre-FEV_1_% predicted**	**post-FEV_1_% predicted**	**pre-** **FEV_1_/FVC**	**post-** **FEV_1_/FVC**

*IREB2*	rs2568494	intron	0.30	0.02*	0.03*	0.05*	0.06
	rs2656069	intron	0.22	0.48	0.29	0.59	0.46
	rs1964678	intron	0.43	0.75	0.91	0.58	0.83
	rs12593229	intron	0.43	0.82	0.79	0.64	0.93
	rs10851906	intron	0.23	0.29	0.17	0.41	0.31
	rs965604	intron	0.43	0.76	0.99	0.54	0.65
	rs13180	exon	0.43	0.69	0.96	0.53	0.79
*LOC123688*	rs8034191	intron	0.29	0.04*	0.07	0.09	0.14
*CHRNA3*	rs1051730	exon	0.31	0.02*	0.03*	0.03*	0.05*

MAF = minor allele frequency

Each model was analyzed assuming an additive mode of inheritance adjusting for pack-years and pack-years^2^

* *p *≤ 0.05

### Interactions with cigarette smoking

There was no association between any of the genotyped SNPs and pack-years of smoking as the outcome in the AAT Genetic Modifiers Study. Inclusion of a SNP-by-pack-years interaction term for the lung function phenotypes showed significant interaction of rs1051730 with pack-years of smoking for the pre- bronchodilator FEV_1_/FVC ratio (main effect; *p *= 0.02, interaction effect; *p *= 0.04). There was no significant association with lung function phenotypes when the study population was stratified into groups of ever-smokers (*n *= 233) and never-smokers (*n *= 145) although this stratified analysis reduced the number of informative families considerably.

### Genotype-by-gender interactions and gender stratification

SNP-by-gender interaction analysis showed significant interaction of rs2568494, rs8034191, and rs1051730 with gender in models for the post-bronchodilator FEV_1_/FVC ratio (main effect; *p *= 0.04, 0.04, 0.02, interaction effect; *p *= 0.02, 0.008, 0.004, respectively). Additionally, rs1051730 showed significant interaction in models for pre and post-bronchodilator FEV_1 _and pre-bronchodilator FEV_1_/FVC ratio (main effect; *p *= 0.04, 0.04, 0.08, interaction effect; *p *= 0.04, 0.02, 0.04, respectively).

In the stratified analysis, for the male subgroup, the *p *values were similar to the whole cohort results, with rs8034191 showing significant association with pre- and post-bronchodilator FEV_1 _percent of predicted and post-bronchodilator FEV_1_/FVC ratio (Table 3). However, in the female subgroup, there was no significant association with lung function phenotypes.

**Table 3:** Genetic association results between SNPs in chromosome 15 and lung function of male subgroup in the AAT Genetic Modifiers Study cohort

*P*-Values for Different Phenotypes						
**Gene**	**SNP**		**pre-FEV_1_% predicted**	**post-FEV_1_% predicted**	**pre-** **FEV_1_/FVC**	**post-** **FEV_1_/FVC**
*IREB2*	rs2568494	intron	0.03*	0.04*	0.07	0.03*
	rs2656069	intron	0.97	0.87	0.64	0.65
	rs1964678	intron	0.99	0.96	0.85	0.78
	rs12593229	intron	0.95	0.93	0.87	0.76
	rs10851906	intron	0.70	0.61	0.84	0.80
	rs965604	intron	0.84	0.90	0.75	0.70
	rs13180	exon	0.85	0.91	0.76	0.71
*LOC123688*	rs8034191	intron	0.04*	0.04*	0.12	0.04*
*CHRNA3*	rs1051730	exon	0.02*	0.02*	0.07	0.02*

Each model was analyzed assuming an additive mode of inheritance adjusting for pack-years and pack-years^2^

* *p *≤ 0.05

### Replication analysis

In the initial analyses in the whole UK dataset, no significant associations with quantitative phenotypes including FEV_1 _and FEV_1_/FVC and qualitative presence of emphysema and COPD were observed (all *p *> 0.05). Gender interaction was apparent for rs2568494 with both COPD and emphysema (main effect *p *= 0.10, interaction *p *= 0.04 and 0.06, 0.03 respectively). No other statistically significant gender interactions were observed. In the sex-stratified models, evidence of association for SNPs in *IREB2 *and *LOC123688 *was observed. A trend was observed for association of rs8034191 and rs2568494 with COPD in the male subgroup, the risk alleles being C and A respectively (both *p *= 0.09). The SNP rs2568494 in *IREB2 *was significantly associated with emphysema in the male subgroup, the A allele conferring an odds ratio and 95% confidence interval (OR and 95% CI) of disease of 2.67 (1.10-6.51, *p *= 0.03). No association was seen with rs1051730 with emphysema.

With addition of a SNP-by-smoking interaction term, both rs8034191 and rs2568494 were associated with COPD in the male subgroup (main effect, *p *= 0.03; interaction effect, *p *= 0.02; and main effect, *p *= 0.04; interaction effect, *p *= 0.003, respectively). Similar associations with emphysema were seen for rs2568494 (*p *= 0.03 and 0.02 respectively). Positive findings in the two datasets are summarized in Table 4.

**Table 4:** Positive findings of genetic association analysis in the AAT Genetic Modifiers Study and the UK AATD National Registry

	AAT Genetic Modifiers Study	UK AATD National Registry
All subjects	Association with pre- and post- FEV_1_% predicted and pre- and post- FEV_1_/FVC	none
Interaction with gender	yes	yes
Interaction with smoking	yes	only in the male subgroup
Male subgroup	association with FEV_1 _and FEV_1_/FVC	association with emphysema association with COPD after adding smoking interaction term

## Discussion

SNPs in the chromosome 15 *CHRNA3/CHRNA5/LOC123688/IREB2 *region have been shown to have associations with lung cancer and COPD unrelated to AAT deficiency. In our current analysis, SNPs in *IREB2, LOC123688 *and *CHRNA3 *genes were shown to be associated with lung function phenotypes in AAT deficient subjects (all PI ZZ) from the AAT Genetic Modifiers Study, and suggested a potential sex-specific effect. Replication in another cohort of AAT deficient subjects from the UK showed that a SNP in *IREB2 *was also associated with emphysema in men. This suggests that chromosome 15q region genes that were found by GWA studies and gene expression analysis of lung tissue samples may also be modifier genes of COPD and emphysema in AAT deficient subjects.

*CHRNA3 *was associated with lung cancer in three separate large GWA studies. This gene was associated with COPD by GWA and the association was replicated in two other COPD cohorts. There have also been recent reports of an association with smoking addiction [27], so it is unclear whether the lung cancer and COPD associations relate to smoking behavior, another aspect of lung biology, or both. *CHRNA3 *is a subunit gene of the nicotinic cholinergic receptor and expressed in autonomic ganglia and brain but is also expressed in bronchial and non-bronchial epithelial cells [28]. Expression in lung cancer cells and signal transduction and apoptosis studies suggests a potential role in carcinogenesis [29]. Interestingly, there are not many observations of lung cancer in patients with AAT deficiency, perhaps because of mortality associated with the development of severe COPD at an early age. Whether there is a common mechanism unrelated to smoking in the pathogenesis of lung cancer and COPD, or whether these previously reported associations relate to smoking addiction is unclear.

IREB2 is a protein of iron-responsive elements (IREs) and is regulated in response to iron and oxygen supply [30]. IREB2-/- mice have aberrant iron homeostasis and accumulate iron in the intestine and the central nervous system(CNS); the CNS accumulation may lead to neurodegenerative disease [31]. Excess iron can be toxic, but the mechanism of neurodegenerative disease is unclear; work is in progress to further characterize the functional pathways impacted by *IREB2 *in the lung. *IREB2 *was found to be differentially expressed according to lung function by microarray experiments, and the SNPs in *IREB2 *showed associations in both a COPD case-control study and family-based studies including the Boston Early-onset COPD and International COPD Genetics Network studies [18]. In a recent report, *IREB2 *polymorphisms were associated with COPD susceptibility in a European population [32]. Interestingly, rs2568494 was significantly associated with COPD in three studies including our current study.

Previous studies of AAT deficient subjects showed that lung function was lower in men than women [33], and previous analyses in the AAT Genetic Modifiers Study also showed lower lung function in men [19]. Our current study suggests that genetic modifier effects of *IREB2 *and *CHRNA3 *may be more prominent in males--potentially contributing to some of the sex-specific features of COPD susceptibility and severity among PI ZZ subjects, although a larger sample size is needed to verify a gene-by-sex interaction.

In this study, there was no association between *IREB2 *and *CHRNA3 *genes and smoking intensity. In the AAT Genetic Modifiers Study, results showed no association when the cohort was stratified by smoking history (ever smokers versus never smokers). However, there was a marginal interaction of rs1051730 with smoking. In the UK study, there were significant smoking interactions of rs2568494 and rs8034191. Smoking markedly increases the risk of COPD and lowers the age-of-onset of COPD in AAT deficient subjects [6,19], and despite small sample sizes, we found reasonable evidence for gene-by-smoking interactions in the chromosome 15q region.

There are several limitations in this study. Multiple statistical comparisons are a potential concern in any complex disease genetics study. Adjusting for either 3 genes or 9 SNPs tested, a *p *value of 0.02 is marginal. Additionally, the association with pulmonary function did not replicate in the UK population, potentially due to phenotypic heterogeneity between the two cohorts. Specifically, the UK subjects have lower mean FEV1 and potentially more emphysema, both of which could influence non-replication. Of note, the association with emphysema was investigated only in the UK population as chest CT scan data collection was not part of the AAT Genetic Modifiers Study. Considering that these SNPs (rs2568494 in *IREB2*, rs8034191 in *LOC123688*, and rs1051730 in *CHRNA3*) were associated with intermediate phenotypes of COPD in other populations and that we include an independent AAT deficient replication cohort, our result are likely meaningful. Additionally, this test- replication approach is even more appealing since all subjects were homozygous recessive for the AAT risk locus (PI ZZ). Also, replication of our results showed association with emphysema, a less heterogeneous pulmonary phenotype. The associated SNPs included two intronic (rs2568494, rs8034191) and one synonymous exonic (rs1051730) SNP. The exonic SNP was not associated with COPD-related phenotypes in the UK cohort. Another limitation of our current study is that rare functional variants in this chromosome 15 region may be contributing to the role of these genes in COPD; genome sequencing efforts in AAT deficient cohorts would be valuable to study rare variant associations. Functional data for associated variants are currently lacking, but many groups are pursuing functional work on this chromosome 15 region.

## Conclusion

We have identified that the chromosome 15q25 region likely contains at least one potential modifier gene of COPD phenotypes in individuals with severe AAT deficiency. The association may be due to smoking behavior, but this is less likely; additionally, these associations may have sex-specific effects. Future directions will include further evaluation of the gene-by-sex interaction in larger cohort with AAT deficiency and identification of the functional variant or variants in this region.

## Abbreviations

AAT: Alpha-1 antitrypsin; CHRNA3: Cholinergic nicotine receptor alpha3; GWA: Genome-wide association; IREB2: Iron regulatory binding protein 2; MAF: Minor allele frequency

## Competing interests

WJK, AMW, AFB, MLB, EJC, EE, GM, RAS and GT have reported that no potential conflicts of interest. SIR was supported from GlaxoSmithKline for travel to meetings for the study. JMS received grant support from Talecris, Baxter. JKS received grant support from AstraZeneca, and honoraria from Talecris, Baxter, CSL Behring, Boehringer Ingelheim, Kamada, Grifols, and has received fees for participation in review activities from Shire and AsthmaTx. CS received consulting fees from AstraZeneca, Talecris, Baxter, Forest, Phamaceuticals, Uptake Medical, Pulmonx and payment for lectures from Talecris and AstraZeneca. EKS received grant support and consulting fees from GlaxoSmithKline for studies of COPD genetics and honoraria and consulting fees from AstraZeneca. RAS received grant support, honoraria and consulting fees and supported for travel to meetings for the study from Talecris. DLD received grant support from Doris Duke Charitable Foundation.

## Authors' contributions

All authors contributed to the study design, data collection and analysis, and writing of the manuscript. WJK, AMW, DLD contributed to data analysis. All authors read and approved the final manuscript.
